# Protective Effect of Honey and Propolis against Gentamicin-Induced Oxidative Stress and Hepatorenal Damages

**DOI:** 10.1155/2021/9719906

**Published:** 2021-09-02

**Authors:** Hassan Laaroussi, Meryem Bakour, Driss Ousaaid, Pedro Ferreira-Santos, Zlatina Genisheva, Asmae El Ghouizi, Abderrazak Aboulghazi, José Antonio Teixeira, Badiaa Lyoussi

**Affiliations:** ^1^Laboratory of Natural Substances, Pharmacology, Environment, Modeling, Health and Quality of Life (SNAMOPEQ), Department of Biology, Faculty of Sciences Dhar Mehraz, Sidi Mohamed Ben Abdellah University, 30000 Fez, Morocco; ^2^CEB-Centre of Biological Engineering, University of Minho, Campus de Gualtar, 4710-057 Braga, Portugal

## Abstract

Bee products are a promising source of phenolic compounds with strong antioxidant activity. The present study was designed to explore the protective effect of honey, propolis, and their combination on gentamicin-induced oxidative stress and hepatorenal dysfunction. This study was conducted on male Wistar rats by intraperitoneal injections of gentamicin (120 mg/kg BW/day, i.p.) or normal saline (1 ml/kg BW/day, i.p.) for 10 consecutive days. Honey (2 g/kg BW), propolis (100 mg/kg BW), or their combination were given daily by gavage to normal and gentamicin groups. Honey and propolis samples were evaluated for their phytochemical composition and antioxidant capacity. The *in vitro* investigations showed that the evaluated samples especially propolis extract have high antioxidant power associated with the presence of several phenolic compounds such as flavonoids, flavan-3-ols, hydroxybenzoic acids, hydroxycinnamic acids, and stilbenes, while honey contains only hydroxybenzoic acids and hydroxycinnamic acids. It was also shown that simultaneous treatment with honey or propolis extract alone or in association prevented changes caused by gentamicin administration and improved hepatic and renal functions. Changes caused by gentamicin administration, observed by *in vivo* experiments, include significant elevation of uric acid, urea, creatinine, and hepatic enzyme levels (ALT, AST, and ALP) and kidney biochemical changes (an increase of urea, uric acid, and creatinine and a decrease of albumin and total protein) as well as remarkable changes of renal and liver oxidative stress markers (CAT, GPx, and GSH) and elevation of MDA levels. Overall, it can be concluded that honey and propolis might be useful in the management of liver and renal diseases induced by xenobiotics.

## 1. Introduction

Aminoglycoside antibiotics, especially gentamicin (GENT), are widely used in the treatment of human and animal bacterial infections, particularly aerobic Gram-negative bacteria. However, the clinical usefulness of gentamicin is limited by its serious side effects on liver and kidney functions [1]. Chronic treatment with GENT prompts tubular necrosis, reduced renal blood flow (RBF), and glomerular filtration rate (GFR) [2]. Previous studies suggest that GENT-toxicity is a complex process in which GENT triggers cellular responses involving various pathways that result in liver and renal injuries [3, 4]. Until the moment, the precise mechanism of GENT hepatonephrotoxicity is not fully understood. Several hypotheses were proposed to explain the involved mechanism of toxicity pathways; oxidative stress is the most reinforced one. Reactive oxygen species (ROS) and other free radicals were considered to be one of the important mediators of GENT-toxicity [5]. *In vivo* and *in vitro* investigations have revealed that GENT stimulates the overproduction of ROS metabolites, leading to necrosis and cellular injury through different pathways, including protein oxidation, lipid peroxidation, and DNA damage. In addition, the excess of free radicals' generation enhances the activation of nitrosative tissue stress, inflammatory markers, modulation of the caspase family, and mitogen-activated protein kinases [6]. It has also been documented that nuclear factor-kappa B (NF-*κ*B), mitogen-activated protein kinase (MAPK), and redox-sensitive transcription factors are associated with GENT-toxicity [7, 8]. Therefore, many researchers are interested in finding new effective and safer antioxidant compounds able to prevent xenobiotic adverse effects.

Since ancient times, honey and propolis were used for the prevention and self-treatment of various illnesses and human disorders [9, 10]. Honey is a functional food produced by bees on mixing plant nectar with its hypopharyngeal excretions [11]. Due to its diverse content of pollen and nectar from different melliferous plants, honey has a wide complex mixture of components. Generally, it contains more than 75% of sugars, around 17% of water, and 3.5% of other minor elements including phenolic compounds, organic acids, proteins, amino acids, enzymes minerals, and vitamins [12]. Owing to this multifaceted composition, honey plays a crucial function in the management of several pathologies such as cancer, inflammation, diabetes, and gastrointestinal dysfunction [13, 14]. Bee glue or propolis is a sticky organic substance produced by honeybees from different exudates and plant buds mixed with wax, pollen, and saliva. It is applied by honeybees as a hive defensive material against various infections. Propolis has been used in folk medicine by different civilizations around the world as a remedy for various illnesses and health disorders [15]. Nowadays, it has become the objective of many scientific investigations. Several reports have documented that propolis extracts possess a broad spectrum of pharmacological actions such as antidiabetic, antimicrobial, and antitumoral effects [15, 16]. Moreover, it was discovered that propolis has a potent protective action in kidney and liver damages [17] which makes it an ideal preventive candidate against GENT adverse effects. It is well documented that the antioxidant capacities of natural products, including beekeeping products, are mostly associated with *in vivo* activities [18]. The assessment of the antioxidant capacity and the quantification of bioactive constituents from honey and propolis by validated and recommended assays are crucial steps to predict their efficacy on oxidative stress and associated pathologies [19, 20]. It has been reported that binary or multiple mixtures of food extracts or their individual bioactive components provide synergy with regard to antioxidant status, anti-inflammation, anticancer, and chemoprevention of several oxidative stress and metabolic disorders [21]. In this context, this study is aimed at assessing the antioxidant potential, identification, and quantification of polyphenols from honey and propolis as well as evaluating their protective effects alone or in combination on GENT-induced oxidative stress and hepatoreno toxicity in Wistar rats.

## 2. Material and Methods

### 2.1. Honey and Propolis Samples and Extract Preparations

Organic honey and propolis sample were collected in July 2018, from modern and healthy hives installed in the Sefrou region (latitude: 33°49′49.89^″^N; longitude: 4°50′7.14^″^W; and altitude: 823 m).

According to our previous survey [22], the predominant vegetation species present around the hives were *Prunus cerasus* L, *Prunus domestica* L, *Ceratonia siliqua* L, *Rosa canina* L, *Olea europaea* L, *Rosmarinus officinalis* L, *Ruta graveolens* L, *Pinus halepensis* Mill, and *Quercus ilex* L.

For experimental assays, propolis extract was obtained with ethanol (70%, v/v). For that one gram of raw propolis was macerated in 30 ml of ethanol-water (70-30, v/v) under mechanical stirring for one week. The final extracts were filtered (Whatman, n°1), and the supernatant was collected. One part of the resulting liquid extract was used for chemical analyses and *in vitro* antioxidant tests. Another part was subsequently concentrated in a rotary evaporator (Model R-200, Büchi labortechnik AG, Switzerland), and distilled water was added to prepare the chosen concentrations for *in vivo* tests. For honey, 2 g of material was dissolved in 10 ml of distilled water and subsequently diluted to prepare the desired concentration. Honey and propolis combination was also studied.

### 2.2. Chemicals

Sodium tungstate, sodium molybdate, phosphoric acid, lithium sulfate, sodium carbonate (Na_2_CO_3_), gallic acid, sodium nitrite (NaNO_2_), aluminum chloride (AlCl_3_), sodium hydroxide (NaOH), sodium carbonate (Na_2_CO_3_), 2,2-Di(4-tert-octylphenyl)-1-picrylhydrazyl (DPPH), 2,2′-azino-bis(3-ethylbenzothiazoline-6-sulfonic acid) diammonium salt (ABTS), potassium persulfate, ferric chloride (FeCl_3_), and all HPLC standard markers (vanillic acid, o-coumaric acid, ferulic acid, ellagic acid, naringin, hesperidin, apigenin, cinnamic acid, resveratrol, rosmarinic acid, rutin, chlorogenic acid, gallic acid, quercetin, and kaempferol) were obtained from Sigma-Aldrich (St. Louis, MO, USA).

### 2.3. Chemical Analyses of Extracts

#### 2.3.1. Total Polyphenol Content (TPC)

Total polyphenols were determined by the Folin-Ciocalteu method following the procedure described by Santos et al. [23]. Briefly, 15 *μ*l of Folin-Ciocalteu reagent sodium tungstate (10 g) and sodium molybdate (2.5 g) was dissolved in 70 ml of distilled water; then, 5 ml of phosphoric acid (85%) and 10 ml of concentrated hydrochloric acid were added. After 10 hours, 15 g of lithium sulfate and 5 ml of distilled water were added, and 100 ml with distilled water and 60 *μ*l of sodium carbonate (Na_2_CO_3_, 75 g/l) were added to 5 *μ*l of honey or propolis extracts. The intensity of the produced color was read at 700 nm by a UV/Vis spectrophotometer (Synergy HT, BioTek Instruments, Inc., USA) after incubating the mixture at 60°C for 5 min. Gallic acid (from Sigma Aldrich, St. Louis, MO, USA) was used as a standard to achieve the calibration curve (2,500-100 mg/l, *R*^2^ = 0.996), and the results were expressed in milligram gallic acid equivalent (GAE) per gram of propolis or honey (mg GAE/g).

#### 2.3.2. Total Flavonoid Content (TFC)

Total flavonoids were determined according to the procedure described by Kong et al. [24]. Briefly, 100 *μ*l of honey or propolis extracts was mixed with sodium nitrite (5%) and 150 *μ*l of aluminum chloride (AlCl_3_) (10%). After 5 min, 200 *μ*l of sodium hydroxide (NaOH) (1%) was added after a 1 h incubation period in the dark. The intensity of the produced color was measured at 510 nm by a UV/Vis spectrophotometer (Synergy HT, BioTek Instruments, Inc., USA). A calibration curve (500-0 mg/l, *R*^2^ = 0.998) was prepared using quercetin (from Sigma Aldrich, St. Louis, MO, USA), and the flavonoid content was expressed as milligrams of the quercetin equivalent per gram of the propolis or honey (mg QE/g).

#### 2.3.3. Identification and Quantification of Polyphenol Compounds by Ultraperformance Liquid Chromatography-Diode Array Detector (UPLC-DAD)

The aqueous solution of honey and ethanolic extract of propolis were analyzed using a Shimadzu Nexera X2 UPLC chromatograph equipped with Diode Array Detector (DAD) (Shimadzu, SPD-M20A, Kyoto, Japan) following the method described and validated by Ferreira-Santos et al. [23]. Separation was performed on a reversed-phase Equity UPLC BEH C18 column (2.1 mm × 100 mm, 1.7 *μ*m particle size; from Waters) and a precolumn of the same material at 40°C. The flow rate was 0.4 ml/min. HPLC grade solvents water/formic acid 0.1% (A) and acetonitrile (B) were used. The elution gradient for solvent B was as follows: from 0.0 to 5.5 min eluent B at 5%, from 5.5 to 17 min linearly increasing from 5 to 60%, and from 17.0 to 18.5 min linearly increasing from 60 to 100%; the column was equilibrated at 5% from 18.5 to 30.0 min. Phenolic compounds were identified by comparing their UV spectra and retention times with that of corresponding standards (vanillic acid (≥97.0% of purity), o-coumaric acid (≥97.0%), ferulic acid (≥99.0%), ellagic acid (≥95.0%), naringin (≥95.0%), hesperidin (≥97.0%), apigenin (≥99.0%), cinnamic acid (≥99.0%), resveratrol (≥99.0%), rosmarinic acid (≥98.0%), rutin (≥94.0%), chlorogenic acid (≥95.0%), quercetin (≥95.0%), kaempferol (≥99.0%), and gallic acid (≥99.0%) are obtained from Sigma Aldrich, St. Louis, MO, USA). Quantification was carried out using calibration curves for each pure compound analyzed using concentrations between 250 and 2.5 mg/l. In all cases, the coefficient of linear correlation was *R*^2^ > 0.99. Compounds were quantified and identified at different wavelengths (209–370 nm). The values of individual phenolic compounds were expressed in milligrams per liter of samples (mg/l). All analyses were made in triplicate.

### 2.4. Evaluation of *In Vitro* Antioxidant Activity

#### 2.4.1. Free Radical Scavenging Activity (DPPH Assay)

Two hundred and seventy microliters of 2,2-diphenyl-1-picryl-hydrazyl-hydrate (DPPH) solution (150 *μ*M, prepared in methanol with an absorbance of 0.700 ± 0.01 at 515 nm) was added to 30 *μ*l of different dilutions of each sample [23]. Then, the mixture reactions were incubated in the dark for 1 h at room temperature. The absorbance was measured at 515 nm, and the antiradical activity (% inhibition) was calculated using Equation (1). DPPH inhibition concentration at 50% (IC_50_) is determined using different dilutions of each sample, considering that the percent inhibition had to be between 20% and 80%, and the results were expressed in milligrams of extract per milliliter (mg/ml). Ethanol 70% and distilled water were used as control solutions instead of the sample.

(1) $$\begin{array}{c} \%\text{inhibition}=\frac{\text{Abs control}-\text{Abs sample}}{\text{Abs control}}\times 100. \end{array}$$

#### 2.4.2. Radical Cation Decolorization (ABTS Assay)

The ABTS assay of honey and propolis extracts was determined as follows: 200 *μ*l of 2,2′-azino-bis (3-ethylbenzothiazoline-6-sulfonic acid) diammonium salt (ABTS) radical cation solution (7 mM of 2,2′-azino-bis(3-ethylbenzothiazoline-6-sulphonic acid) diammonium salt dissolved in ultrapure water containing 2.45 mM of potassium persulfate) was mixed with 10 *μ*l of different dilutions of each extract. The resulting solutions were incubated in the dark for 30 min at room temperature, and the intensity of produced color was measured immediately at 734 nm by a UV/Vis spectrophotometer (Synergy HT, BioTek Instruments, Inc., USA). The ABTS radical cation inhibition percent was determined using Equation (1) [23]. The IC_50_ results were expressed in milligrams per milliliter.

#### 2.4.3. Ferric Reducing Antioxidant Power (FRAP Assay)

Two hundred ninety microliters of FRAP reagent (10 mM of 2,4,6-tri(2-pyridyl)-s-triazine solution (made in 40 mM HCl) mixed with 20 mM of ferric chloride (FeCl_3_) and 0.3 M acetate buffer (pH 3.6) in a proportion of 1 : 1 : 10 (v/v/v)) was mixed with a 10 *μ*l aliquot of honey and propolis extract, and the absorbance was determined at 593 nm by a UV/Vis spectrophotometer (Synergy HT, BioTek Instruments, Inc., USA) after the incubation of the reaction mixture in the dark at 37°C for 15 min [23]. The aqueous solution of ferrous sulfate FeSO_4_.7H_2_O (1000–100 *μ*M) was used for standard curve preparation (*R*^2^ = 0.993). The FRAP values are expressed as millimoles of ferrous equivalent per gram of samples (mmol Fe^2+^/g).

### 2.5. Experimental Animal's Protocol

Forty-two male Wistar rats weighing 165.42 ± 5.8 g, obtained from the Animal Housing Breeding Center, Department of Biology, Faculty of Sciences Dhar El Mahraz, University Sidi Mohamed Ben Abdallah, Fez, Morocco, were used for these experiments. Rats were kept in a ventilated room and lived in standard environmental conditions (22 ± 3°C, 55 ± 5% humidity, and 12 h light/dark cycles). The present work was designed under ethical approval number (USMBA-SNAMOPEQ 2017-03), certified by Sidi Mohamed Ben Abdellah University, Fez, under the responsibility of the Animal Facility and the Laboratory of Natural Substances, Pharmacology, Environment, Modeling, Health and Quality of Life. The manipulation of animals respected the EU Directive 2010/63/EU for animal experiments to avoid and minimize animal suffering and the number of experimented animals.

Rats were randomly divided into 7 experimental groups of 6 rats.

*Group DW (control group)*: received daily by gavage distilled water and injected with normal saline solution (1 ml/kg BW/day, i.p.).

*Group PR (propolis group)*: received daily by gavage propolis extract (100 mg/kg BW) and injected with normal saline solution (1 ml/kg BW/day, i.p.).

*Group H (honey group*): received daily by gavage honey (2 g/kg BW) and injected with saline solution (1 ml/kg BW/day, i.p.)

*Group Gent (gentamicin group)*: received daily by gavage distilled water and injected with GENT prepared in saline solution (120 mg/kg BW/day, i.p.)

*Group Gent+PR (gentamicin/propolis group)*: received daily by gavage propolis extract (100 mg/kg BW) and injected with GENT (120 mg/kg BW/day, i.p.)

*Group Gent+H (gentamicin/honey group)*: received daily by gavage honey (2 g/kg BW) and injected with GENT (120 mg/kg BW/day, i.p.)

*Group Gent+Pr+H (gentamicin/propolis/honey group)*: received daily by gavage 100 mg/kg BW of propolis extract +2 g/kg BW of honey and injected with GENT (120 mg/kg BW/day, i.p.)

Rats of all groups received a normal chow diet (carbohydrate (48%), protein (21%), fat (3%), fiber (5%), calcium (0.8%), phosphorus (0.4%), moisture (13%), and ash (8%)) twice a day.

The study lasted for 10 days. At the end of the experiment, rats underwent fasting for 12 hours after their last feeding; then, urine samples were collected using specific metabolic cages, and blood samples were taken into tubes from each rat by retroorbital bleeding under ether anesthesia, and then, plasma was separated by centrifugation (2000 g) during 10 min.

The treatment duration was chosen according to [25]. Honey and propolis extract doses were selected based on the studies of El-Haskoury et al. and El Menyiy et al. [26, 27].

Honey and propolis were prepared every single day one hour before their administration at a concentration of 2 g of honey for 10 ml and 100 mg of dry propolis extract for 10 ml. The combination of both samples was performed by mixing 2 g of honey and 100 mg of propolis dry extract in 10 ml of distilled water. The combined mixture is vortexed throughout the gavage period.

### 2.6. Biochemical Analysis

After 10 days of treatment, urine and blood samples (plasma) were collected for the analyses of different kidneys and liver biomarkers: urea (kit number 7D75-30, urease/NADH method), uric acid (kit number 7D76-20, Uricase/POD method), creatinine (kit number 7D64-20, picric acid/NaOH method), albumin (kit number7D53-20, bromocresol Green method), total protein (kit number 7D73-20, biuret method), aspartate aminotransferases (AST) (kit number 7D81-20, aspartate/NADH method), alanine aminotransferases (ALT) (kit number 7D56-20, alanine/NADH method), alkaline phosphatase (ALP) (kit numbers 7D55-20 and 7D55-20, colorimetric method), and lactate dehydrogenase (LDH) (kit number 7D69-20, lactate/NAD method). C-reactive protein (CRP) was estimated using kit numbers 6k26-30 and 6K26-41, immunoturbidimetry method (Architect c8000i biochemistry analyzer) [28].

### 2.7. Liver and Kidney Antioxidant Enzyme Activities

Catalase (CAT) activity was calculated according to the method of [29]. A decrease in absorbance due to H_2_O_2_ degradation was monitored spectrophotometrically at 240 nm for 1 min, and the activity was expressed as *μ*mol H_2_O_2_/min/mg protein. Glutathione peroxidase (GPx) activity was estimated according to the method of [30]. The activity was expressed as moles of GSH oxidized/min/mg protein.

### 2.8. Reduced Glutathione (GSH)

GSH levels were measured following the protocol described by Ellman [31]. Briefly, 3 ml of sulfosalicylic acid (4%) was added to 500 ml of homogenate liver and kidney tissues. The mixture was centrifuged at 2,500 × g for 15 min, and then prepared Ellman's reagent was added to 500 ml of supernatant. The absorbance was measured at 412 nm after 10 min. Total GSH content was expressed as micrograms per milligram of protein.

### 2.9. Lipid Peroxidation (TBARS)

The formation of products of lipid peroxidation was quantified in liver and kidney tissues using the thiobarbituric acid-reactive substances (TBARS) method, as reported previously by Kassan et al. [32], and absorbance was measured at 532 nm. Results were expressed as malondialdehyde (MDA) concentration (nmol/g tissue).

### 2.10. Statistical Analysis

All data are presented as mean ± SD (standard deviation). Statistical comparisons between the groups were performed with a one-way analysis of variance (ANOVA) followed by the Tukey test. Chi-square was used to compare the difference in the proportion of the effect of gentamicin, honey, propolis, or both of them on oxidative parameters between kidney and liver. The GraphPad Prism® software (version 5.0; GraphPad Software, Inc., San Diego, USA) was used, and *p* < 0.05 was considered significant.

## 3. Results

### 3.1. Phytochemical Analysis and *In Vitro* Antioxidant Activities of Honey and Propolis Extracts

As shown in Table 1, propolis presented potent antioxidant activity. More precisely, propolis extract contains 76.00 ± 2.15 mg GAE/g of total polyphenol content and 42.22 ± 0.96 mg QE/g of total flavonoid content. However, total polyphenol and flavonoid contents in honey are 2.08 ± 0.04 mg QE/g and 0.74 ± 0.01 mg QE/g, respectively. The mixture of honey and propolis showed 73.98 ± 4.27 mg GAE/g of total polyphenols and 44.01 ± 0.13 mg QE/g of flavonoids. Regarding the IC_50_ of DPPH assay, propolis, honey, and their mixture showed values of 0.15 ± 0.01 mg/ml, 9.78 ± 0.95 mg/ml, and 0.13 ± 0.02 mg/ml, respectively. For ABTS antioxidant test, the IC_50_ values are 0.08 ± 0.02 mg/ml for propolis, 4.91 ± 0.12 mg/ml for honey, and 0.41 ± 0.05 mg/ml for honey+propolis mixture, while the values of FRAP test were 2.15 ± 0.04 mmol Fe^2+/^g for propolis, 0.025 ± 0.00 mmol Fe^2+/^g for honey, and 2.27 ± 0.09 mmol Fe^2+^/g for their mixture.

### 3.2. Phenolic Compound Quantification from Honey and Propolis Extracts

UHPLC-DAD analysis revealed the presence of several phenolic compounds belonging to the following groups: phenolic acids, flavonoids, flavanols, flavanones, and stilbenes. As shown in Table 2, thirteen phenolic compounds were quantified in propolis extract and six were determined in honey solution. Rosmarinic acid (470.35 ± 52.0 mg/l), hesperidin (417.18 ± 50.0 mg/l), and resveratrol (116.89 ± 12.7 mg/l) were the most abundant phenolic compounds detected in hydroethanolic propolis extract. Other detected components in propolis extract are vanillic acid, o-coumaric acid, ferulic acid, ellagic acid, naringin, apigenin, rutin, chlorogenic acid, quercetin, kaempferol, and gallic acid. However, gallic acid (30.06 ± 0.23 mg/l) was the predominant compound found in honey followed by ferulic acid (8.35 ± 0.01 mg/l), chlorogenic acid (7.06 ± 0.11 mg/l), ellagic acid (5.09 ± 0.02 mg/l), cinnamic acid (4.25 ± 0.01 mg/l), and finally vanillic acid (2.90 ± 0.01 mg/l).

### 3.3. Effect of Honey and Propolis Extracts on Renal Biochemical Changes

**Figure 1:**
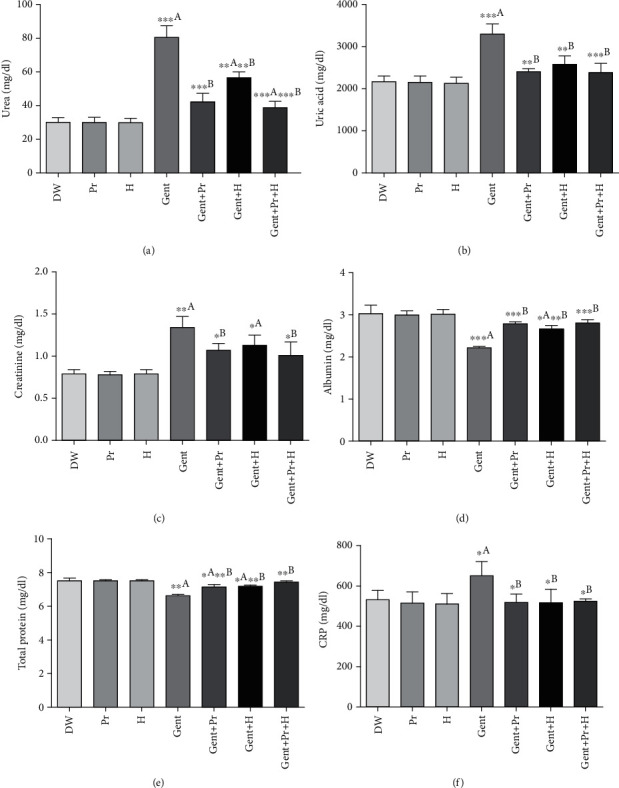
Effect of propolis, honey, or their combination on gentamicin-induced changes in (a) plasmatic urea, (b) uric acid, (c) creatinine, (d) albumin, (e) total protein, and (f) CRP in Wistar rats. The results were presented as mean ± SD of 6 rats; DW: distilled water; Pr: propolis; H: honey; Gent: gentamicin. ^a^Comparison between the DW group and all groups; ^b^comparison between the Gent group and gentamicin cotreated groups.

**Figure 2:**
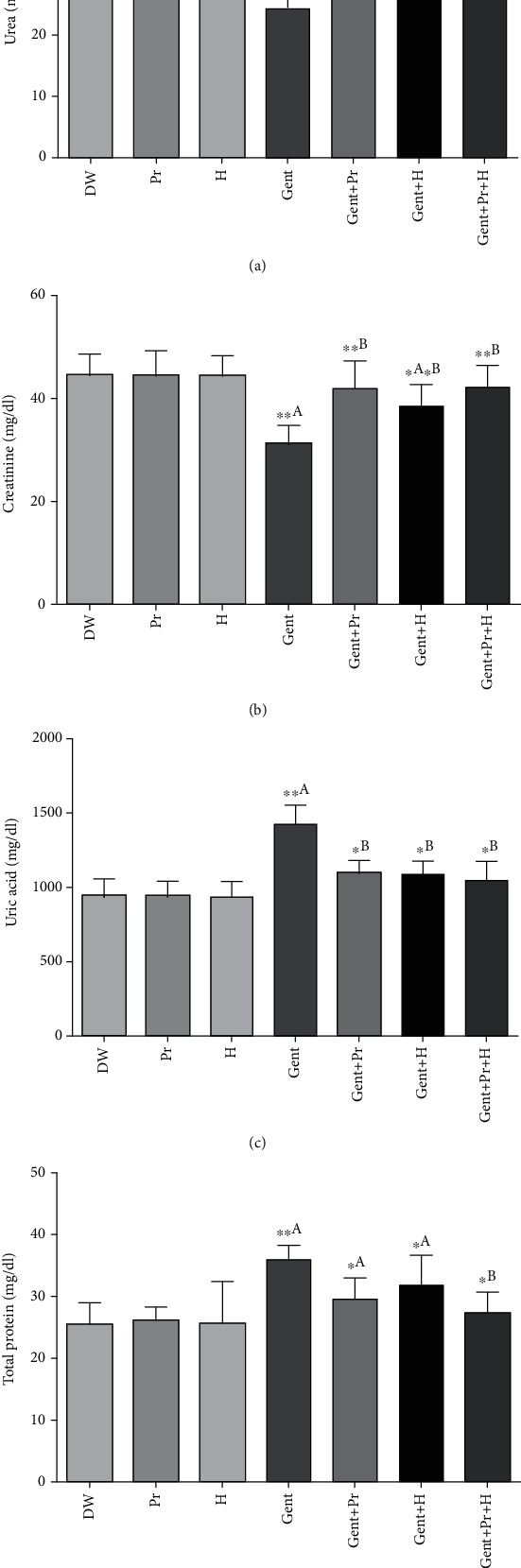
Effect of propolis, honey, or their association on gentamicin-induced changes in (a) urinary urea, (b) uric acid, (c) creatinine, and (d) total protein in Wistar rats. The results were presented as mean ± SD of 6 rats; DW: distilled water; Pr: propolis; H: honey; Gent: gentamicin. ^a^Comparison between the DW group and all groups; ^b^comparison between the Gent group and gentamicin cotreated groups; ^d^comparison between the Gent+H group and the Gent+Pr+H group. ^∗^*p* < 0.05, ^∗∗^*p* < 0.01, and ^∗∗∗^*p* < 0.001.

The data in Figure 1 indicates that gentamicin administration (120 mg/Kg/BW) for 10 consecutive days increased plasmatic levels of urea, uric acid, creatinine, and CRP as well as decreased plasma albumin and total protein levels as compared to the control group (DW). The cotreatment with propolis extract alone (100 mg/Kg/BW) or honey alone (2 g/Kg/BW) or both (propolis+honey) significantly prevented the increase of plasma urea, uric acid, creatinine, and CRP levels and increased plasma albumin and total protein levels in comparison with group Gent. In addition, GENT injection induced a significant elevation in urinary uric acid and total protein levels, while showed a decrease in urea and urinary creatinine compared to the control group (DW). However, its coadministration with propolis, honey, or their combination attenuated the deleterious effects of GENT showed a significant decrease in urinary uric acid and total protein levels accompanied by a higher level of urinary urea and creatinine comparatively to the Gent group (Figure 2). Propolis and honey showed no (negative) effects on the kidney of normal rats.

### 3.4. Effect of Honey and Propolis Extracts on Liver Biochemical Changes

**Figure 3:**
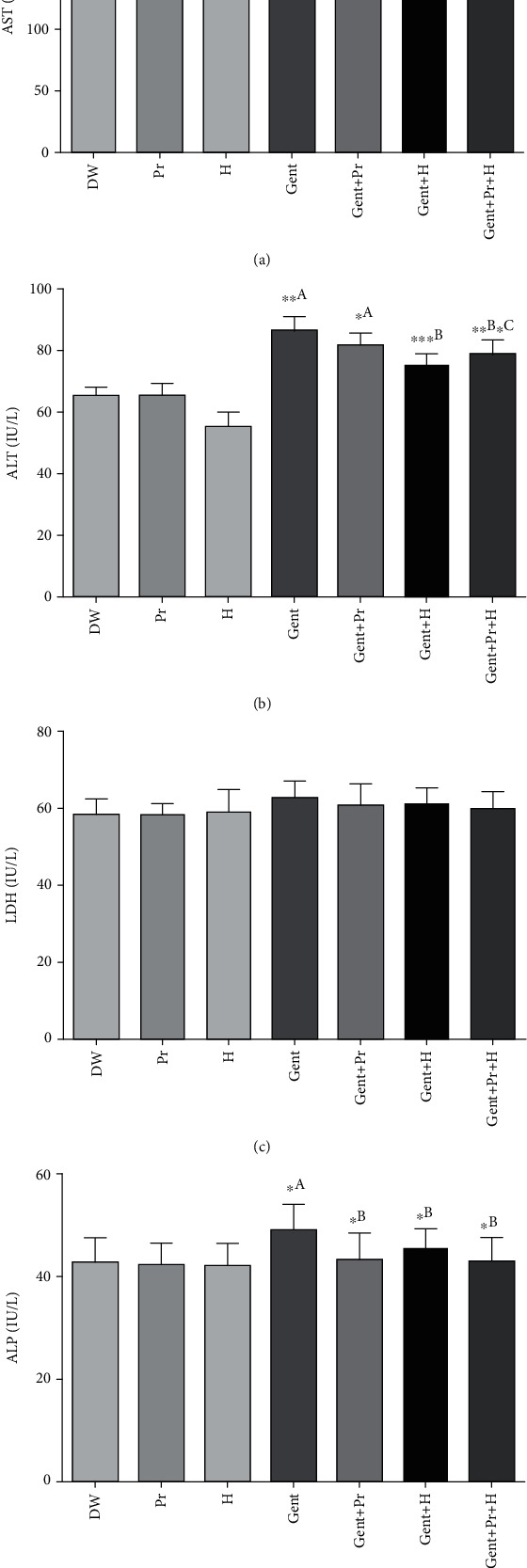
Effect of propolis, honey, or their combination on gentamicin-induced changes in (a) aspartate aminotransferases (AST), (b) alanine aminotransferases (ALT), (c) lactate dehydrogenase (LDH), and (d) alkaline phosphatase (ALP) in Wistar rats. The results were presented as mean ± SD of 6 rats; DW: distilled water; Pr: propolis; H: honey; Gent: gentamicin. ^a^Comparison between the DW group and all groups; ^b^comparison between the Gent group and gentamicin cotreated groups; ^c^comparison between the Gent+Pr group and the Gent+Pr+H group; ^d^comparison between the Gent+H group and the Gent+Pr+H group. ^∗^*p* < 0.05, ^∗∗^*p* < 0.01, and ^∗∗∗^*p* < 0.001.

Regarding hepatic function, GENT treatment (group Gent) induced a significant increase in enzymatic levels of AST, ALT, and ALP as compared to the normal group (DW), while no significant change was observed in plasma levels of LDH between studied groups (Figure 3), whereas the cotreatment with propolis (Gent+PR), honey (Gent+H), or their combination (Gent+PR+H) showed a preventive effect in the increase of plasmatic AST, ALT, and ALP levels promoted by GENT administration. Propolis and honey did not show negative effects on liver normal rats.

### 3.5. Effect on Kidney Oxidative Stress

GENT administration induced marker disorganization of oxidant status and proteins in the kidney tissue, as characterized by a significant reduction in CAT, GSH, GPx, and proteins with a concomitant elevation in MDA concentration compared to the DW group (Table 3). However, the coadministration of propolis alone, honey alone, or their association significantly lowered the MDA levels and prevented the decrease of CAT, GSH, and GPx, activities, and protein levels as compared to the GENT group. These results demonstrated that propolis and honey administrations especially their combination improve the kidney state and exhibit a significant nephroprotective effect by restoring the antioxidant capacity and attenuating the oxidative stress in the kidney.

### 3.6. Effect on Liver Oxidative Stress

Regarding the liver tissue, GENT treatment (group Gent) caused a remarkable reduction in CAT, GSH, and GPx activities (*p* < 0.01) and induced a decrease in protein content (*p* < 0.05) associated with a high elevation in MDA (Table 4) in comparison with the nontreated group (group DW), while the cotreatment with propolis alone, honey alone, or their combination significantly prevented the increase of MDA levels and promotes beneficial effects on antioxidant enzyme activities, as well in protein levels as compared to the GENT group.

### 3.7. Comparison between Kidney and Liver Oxidative Stress

Regarding the effect of interventions on the kidney and liver tissues, GENT caused a remarkable decrease in the CAT (40.31 vs. 24.67%, *p* = 0.001), GSH (57.80 vs. 54.52%, *p* = 0.005), and a significant increase in the MDA (80.27 vs. 38.27%, *p* = 0.001) levels of the kidney in comparison with the liver tissue. Also, GENT administration induced a significant decrease in liver protein compared to the kidney (39.44 vs. 28.58%, *p* = 0.002). The coadministration of propolis (GENT+PR) significantly increased CAT, GSH, and GPx activities (52.10 vs. 27.15%, *p* = 0.001; 111.87 vs. 80.78%, *p* = 0.001; and 46.46 vs. 36.18%, *p* = 0.002, respectively) and moderately decreased MDA levels (26.61 vs. 18.87%, *p* = 0.009) in the kidney as compared to the liver tissue. Likewise, the coadministration of propolis remarkably increased the protein level in the kidney tissue liver (53.27 vs. 37.57%, *p* = 0.001). The cotreatment with honey (Gent+H) showed a significant elevation in the kidney CAT (32.36 vs. 23.58%, *p* = 0.001), GSH (99.00 vs. 87.31%, *p* = 0.003), GPx (34.65 vs. 21.56%, *p* = 0.003), and protein (72.08 vs. 50.29%, *p* = 0.001) as well as expressed a significant decrease in MDA (23.82 vs. 18.00%, *p* = 0.031) in comparison to their levels in the liver tissue. The combined cotreatment (Gent+PR+H) increased significantly the levels of CAT (58.29 vs. 29.32%, *p* = 0.001) and GSH (122.54 vs. 97.80%, *p* = 0.001) in the kidney as compared to the liver tissue as well as increased the lower level of proteins (57.09 vs. 41.92%, *p* = 0.001) and GPx activity in the liver tissue than in the kidney tissue (50.18 vs. 35.79%, *p* = 0.001) (Table 5).

### 3.8. The Effect of Propolis, Honey, or Their Combination on the Gentamicin-Induced Body and Organ Weight Changes

As shown in Table 6, gentamicin injection induced a marked reduction in body weight gain accompanied by a significant increase in the relative kidneys and liver weights. However, the treatment with propolis (Gent+Pr), honey (Gent+H), or their mixture (Gent+Pr+H) prevented the body-weight loss and improved the relative organs' weights.

## 4. Discussion

### 4.1. Phytochemical Constituents and Antioxidant Activities of Honey and Propolis Extracts

The identification and quantification of bioactive components of natural products are crucial tests to better understand the pharmacological effects and their effectiveness. Flavonoids and other phenolic compounds are powerful antioxidant molecules with a stronger ability to fight free radicals that induce oxidative stress [33]. DPPH, ABTS, and FRAP are the most commonly used tests to evaluate the antioxidant activities of various biological matrices, evaluating different mechanisms of action. The present study illustrates that propolis has a more affluent profile of antioxidant phenolic compounds when compared to honey alone or mixed with propolis extract. Polyphenol content in propolis extract was 76.00 ± 2.15 mg GAE/g, and the value obtained was higher than the data reported by Touzani et al. [34] for propolis sample harvested from the same locality where our evaluated sample was collected (Sefrou). The flavonoid content (42.22 ± 0.96 mg QE/g) was higher than those obtained by Miguel et al. [35] for Moroccan propolis, where the values ranged from 0.2 to 34.3 mg QE/g. The amount of antioxidant (flavonoids and nonflavonoids) compounds in propolis depends strongly on the botanical origin of resin and pedoclimatic characteristics of the collecting region, which explain the wide fluctuation between the evaluated sample and other propolis samples from different localities [18, 36]. Regarding antioxidant activity, the propolis extract concentration required to inhibit 50% of DPPH was 0.15 ± 0.01 mg/ml. Data are in line with those reported by Miguel et al. [35] who studied fourteen propolis samples harvested from different ecoregional origins in Morocco, with IC_50_ values oscillated between 0.025 mg/ml and 1.813 mg/ml. ABTS and FRAP activities were 0.08 ± 0.02 mg/ml and 2.15 ± 0.04 mmol Fe2+/g, respectively. The FRAP value was higher than those signaled by Svečnjak et al. [37] for Adriatic Sea islands propolis, ranging between 0.1 and 0.8 mmol Fe^2+^/g.

Concerning honey, the polyphenol content was 2.08 ± 0.04 mg GAE/g, which was higher than those reported by Petretto et al. [38] for teen Moroccan monofloral honey. Regarding antioxidant activities, the concentration of honey necessary to inhibit 50% of DPPH radical was 13.53 ± 1.3 mg/ml, and this value was similar to the results of Laaroussi and coworkers [11] reported on eight Moroccan honey samples, varied from 13.54 to 45.34 mg/ml and higher than those signaled for the Malaysian honey ranging from 6.12 to 11.56 mg/ml [39]. There is a strong correlation between the pollen profile of different honey samples and their amount in phenolic and flavonoid compounds [11]. In addition to the floral origin, the wide variation seen between the antioxidant compounds of examined sample and those reported by other investigations is attributed to many other factors including harvest season and geographical and environmental characteristics of the areas where it is produced [40]. ABTS and FRAP activities were 4.91 ± 0.12 mg/ml and 0.025 ± 0.00 mmol Fe^2+^/g, respectively. The ABTS and FRAP values of analyzed honey were found to be lower than those reported by Kıvrak and Kıvrak [41] for Turkish honey, where ABTS values are oscillating from 8.22 to 41.20 mg/ml and by Mahmoodi-Khaledi et al. [42] where FRAP values are ranging between 0.027 and 0.182 mmol Fe^2+^/g. It is well documented that *in vitro* interactions between phytobioactive components induce changes in overall antioxidant capacities [43]. The mixture of honey and propolis extract showed a higher amount of phenolics and flavonoids and displayed strong DPPH radical scavenging capacity, ABTS scavenging capacity, and high reducing power antioxidant activity (FRAP) as compared to honey alone and revealed higher antioxidant activities (DPPH and FRAP) as compared to propolis extract alone (Table 1). Substantially, the examined extracts (alone or in the mixture) showed powerful antioxidant capacities, which are possibly related to their individual antioxidant components mainly flavonoids, phenolic acids, and terpenoids as well as to the possible interaction between them. Skroza and coworkers have examined the antioxidant effect of gallic acid, caffeic acid, quercetin, catechin alone, or in combination with resveratrol using three different antioxidant assays (DPPH, FRAP, and Briggs–Rauscher reaction) [44]. The obtained results showed better antioxidant capacities of evaluated compounds when tested in the mixture form as compared with the activities displayed when testing individual compounds, which proves the possible interaction between these bioactive components leading to synergistic antioxidant effects.

### 4.2. Effect of Interventions on Renal Function

#### 4.2.1. Effect on Kidney Biochemical Changes

Nowadays, infectious diseases are a challenging public health problem worldwide, and face to bacterial resistance and antibiotics overuse including GENT may prompt side effects such as hepatorenal toxicities [45].

The present data showed that GENT (120 mg/kg BW) caused kidney dysfunction demonstrated by a significant elevation in blood levels of urea, uric acid, and creatinine and a remarkable decrease in plasmatic albumin and total protein levels. In addition, GENT administration increased urinary uric acid and protein levels and decreased urea and creatinuria levels, which are classified as a major sign of renal tubular necrosis and kidney damage [46]. These results are similar to the data reported by Marinho et al. [47]. A study published by Al Za'abi et al. [48] showed that GENT reduces the ability of adenosine triphosphate (ATP) in the renal tubular cells by inhibiting its phosphorylation process, which leads to low membrane integrity and thus the sensitivity to the overproduction of free radicals [49]. The animals submitted to GENT injection and cotreated with propolis alone (Gent+PR), honey (Gent+H), or their association (Gent+PR+H) restored all investigated plasmatic and urinary biomarkers. Similar data have been reported by Aldahmash and coworkers [50]. The pharmacological effects of functional organic products are most often linked to their phenolic compound composition and their ability to counteract free radicals [51]. *In vitro* investigations showed that honey and propolis extracts contain several bioactive molecules including rosmarinic acid, resveratrol, hesperidin, and gallic acid. Tavafi and coworkers have reported that rosmarinic acid exerts its renoprotective action by inhibiting lipid peroxidation and upregulating renal GSH, GPx, and CAT activities as well as by decreasing renal MDA levels [52]. Moreover, rosmarinic acid was found to protect kidney damage caused by cisplatin through the downregulation of tumor suppressor p53 and p-p53 and reduction of cleaved caspase-3, signifying the inhibition of tubular apoptosis [53]. As a part of its nephroprotective action, resveratrol has been revealed to inhibit the expression of transforming growth factor-beta (TGF-*β*), transforming growth factor-beta receptor1 (TGF-*β*R1), and Smad3 that play a key role in kidney fibrosis [54].

#### 4.2.2. Effect on Kidney Oxidative Parameters

It has been proven that overproduced ROS induces oxidative stress and organ damage. In this study, GENT administration deteriorates the renal antioxidant defense system and increases oxidative biomarkers. Rats injected with 120 mg/kg of GENT without cotreatment for 10 consecutive days expressed low CAT, GSH, and GPx activities and high levels of renal MDA. Results obtained from the current study are consistent with the findings of Edeogu et al. [55] in which GENT administration (100 mg/kg BW) markedly increased renal MDA levels and significantly depressed renal activities of GSH, SOD, CAT, and GPx levels in kidney tissues.

**Figure 4:**
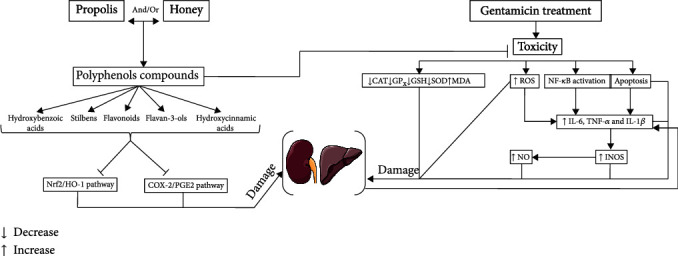
Proposal mechanism of action of honey and propolis against hepatorenal toxicity and oxidative stress induced by gentamicin.

It was suggested that GENT may trigger the release of renal mitochondrial iron to form a Fe–GENT complex which promotes the overproduction of ROS and other free radicals such as H_2_O_2_ [56]. The generated H_2_O_2_ enhances the production of superoxide anions (O_2_^•-^) in the renal mitochondrial cortex [57]. In the presence of redox metal ions, hydrogen peroxide (H_2_O_2_) and superoxide ion (O_2_^•-^) generate hydroxyl radical (HO^•^) which is considered as one of the most damaging ROS [58]. Moreover, [59] found that GENT enhances the overexpression of proinflammatory cytokines such as interleukin-6 (IL-6) and tumor necrosis factor-*α* (TNF-*α*) which largely contributes to tissue injury and leads to the development of various complications including nephrotoxicity. Likewise, GENT overuse leads to mitochondrial dysfunction and promotes the excessive generation of free radicals, which are considered as potent oxidizing agents that elicit lipid proteins and DNA oxidative damage via the upregulation of NADPH oxidase 4 (NOX4) and the suppression of adenine nucleotide translocase 2 (ANT2) [60–63]. Accordingly, the chronic accumulation of oxidative DNA injuries may enhance mutagenesis and human pathogenesis [64]. Interestingly, the coadministration of honey, propolis, or their association had significantly increased the activities of antioxidant enzymes and prevents the increased level of MDA in the kidney, which confirms their nephroprotective ability. These results were in agreement with the findings of [26], in which Moroccan carob honey increased CAT, GSH, SOD, and GPx activities and ascorbic acid as well decreased MDA levels in renal and liver tissues of rats treated with carbon tetrachloride (CCL4). In the same context, the results of Shi et al. showed that propolis improves streptozotocin-induced renal dysfunction and oxidative stress by inhibiting lipid peroxidation and ameliorating antioxidant system defenses [65]. Owing to its health-promoting compounds, honey inhibits the production of proinflammatory cytokines NOx, IL-6, TNF-*α*, MCP-1, IL-12p70, IFN-*γ*, and IL-10 and attenuates nuclear factor-kappa B (NF-*κ*B) translocation to the nucleus [66]. Due to their rich composition in bioactive molecules, honey and propolis might have renoprotective action through one or different signaling pathways (Figure 4). Indeed, gallic acid, a hydroxybenzoic acid present at a high concentration in honey, displays its nephroprotective effects via the modulation of oxidative stress and inhibition of inflammatory response [67]. Moreover, hesperidin, a flavonoid present in bee products, prevents kidney damage by inhibiting the cyclooxygenase-2/prostaglandin E2 (COX-2/PGE2) signaling pathway [68].

Overall, the nephroprotective effect of investigated honey and propolis extracts could be due to the interaction between their bioactive components through diverse signaling pathways (Figure 4).

### 4.3. Effect of Interventions on Liver Function

#### 4.3.1. Effect on Liver Biochemical Changes

The current findings showed that GENT-treated rats exhibited extreme liver damage manifested by a remarkable increase in plasma levels of ALT, AST, and ALP (Figure 3). These results were similar to those reported by Hegazy et al. [69]. The high plasmatic activity of the examined enzymes reflects hepatic cell necrosis and liver structural changes, which leads to their secretion into the bloodstream from the cytosol [70]. However, GENT animals cotreated with propolis, honey, or their association expressed a significant decrease in plasmatic AST, ALT, and ALP levels, which indicate that honey and propolis extract especially their combination blocked the enzyme leakage and prevented GENT-caused kidney dysfunction. These results go on hand with the findings of El-Haskoury et al. [26] who reported that honey exhibited strong hepatoprotective action against CCL4-induced toxicity. Likewise, propolis extract (150 mg/kg BW) was found able to prevent kidney injury and renal dysfunction induced by aluminum chloride in rats [17].

#### 4.3.2. Effect on Liver Oxidative Parameters

In addition to the biochemical investigations, the assessment of liver oxidative stress showed that GENT treatment decreased significantly CAT, GSH, and GPx activities, as well as protein levels with a concomitant increase in liver MDA level, a major sign that the organ is functionally compromised, with an imbalance of its redox state. It is well reported that liver tissue is rich in polyunsaturated fatty acids (PUFAs), which are sensitive to peroxidative damage [71]. Alterations in gene expression profiles resulting from chronic exposure to xenobiotic substances have a negative impact on human health [72]. A previous study showed that CCL4 treatment downregulated the mRNA expression of SOD, GPx, and CAT, which was reversed by *Actiniopteris radiata* active compounds in a dose-dependent manner [73]. The suppressed activity of these antioxidant enzymes may be due to the interruption of their synthesis by the overproduced ROS and other free radicals, which could be the same mechanism involved following the GENT treatment. Additionally, it has been documented that total protein and globulin levels were correlated positively with SOD and GPx activities of rats treated with curcumin against sodium salicylate-induced oxidative liver and kidney damages [74].

However, rats receiving GENT and simultaneously treated with honey, propolis, or their combination expressed lower MDA levels and higher CAT, GSH, GPx activities and protein contents. Similar data have been documented by other researchers [75, 76]. In a human trial study, Diniz et al. have reported that the administration of microencapsulated standardized Brazilian green propolis extract (375 and 750 mg/day) during 7 days rose the endogenous enzymatic and nonenzymatic antioxidants and reduced the biomarkers associated with the cellular membrane and DNA damage in healthy young participants [77]. Similarly, Ebeid and coworkers have demonstrated that propolis supplementation (400 mg, 3 times daily) for 10 consecutive days before and 10 days after the radiotherapy session had been able to reduce significantly the DNA oxidative damage of breast cancer patients [78]. This effect was expected since several propolis components could promote the DNA repair processes, which in turn scavenge the free radicals generated by chemotherapeutic agents and counteract the impairment caused by infrared radiations [79]. *In vitro*, similar results were documented by Haza and Morales [80] in which polyfloral honey improved the antioxidant defense system and protected HepG2 cells against different food mutagen-induced DNA oxidative damage.

The hepatoprotective action of these bioactive products could be attributed to their antioxidant content which assists in the preservation of membrane integrity. Honey and propolis extracts contain a variety of natural antioxidants, such as flavonoids, flavan-3-ols, hydroxybenzoic acids, hydroxycinnamic acids, and stilbenes (Table 2), which make them functional foods with a broad spectrum of pharmacological activities. The antioxidative properties of phenolic components are related to several mechanisms of action including, singlet oxygen quenching, free radical scavenging, metal ion chelation, and hydrogen donation [81]. Previous works documented that phenolic compounds possess several biological activities and numerous beneficial health effects. The outcome of Mahmoud and coworkers [82] showed that ferulic acid prevents oxidative stress and exerts its hepatoprotective activity against methotrexate-induced hepatotoxicity through the upregulation of nuclear factor erythroid 2-related factor/heme oxygenase-1 (Nrf2/HO-1) signaling pathway. Gallic and hydroxybenzoic acids present in our studied samples prevent liver injury by modulating the expression and phosphorylation of epidermal growth factor receptor (EGFR) [83]. A previous study reports that kaempferol has been revealed to exert a potent hepatoprotective effect by inhibiting cytochrome P-450 2E1 (CYP2E1) and thus reducing ROS production [84]. In the same context, chlorogenic acid, an active phenolic compound found in bee products, has been already documented to have potent antiradical action and high preventive ability on arsenic-induced oxidative stress and apoptosis [85]. The food synergy notion, which assumes that different dietary constituents have additive or even synergistic effects on human health, has been previously pointed out [21]. As found in nature, bioactive compounds are usually present in mixture form, which increases the possibility of interactions between them. The combination of food extracts or their bioactive compounds may enhance the *in vitro* antioxidant status and promote the *in vivo* antioxidants/ROS ratio [86]. For instance, a binary combination of *α*-tocopherol with kaempferol or myricetin showed a better preventive effect on free radicals inducing oxidative stress and lipid peroxidation than each component alone [87]. Likewise, rats fed a diet supplemented with a mixture of quercetin (30 mg/kg/day) and resveratrol (15 mg/kg/day) synergistically reduced the fat accumulation in the white adipose tissue and improve triacylglycerol metabolism [88]. Moreover, Baranowska and coworkers have reported that quercetin combined with naringenin improves synergistically cellular redox status and reduces global DNA methylation [89].

Overall, the hepatoprotective action documented by our examined samples could be related to molecular interactions between their individual bioactive constituents through one or several signaling pathways (Figure 4).

### 4.4. Effect of Interventions on Body and Organ Weight Changes

The rats' weights were measured at the beginning and the end of the experiment. Gentamicin treatment (120 mg/kg BW/day, i.p.) exhibited a significant reduction in body weight gain as compared to the normal untreated rats (DW). The documented body weight loss was in agreement with the findings of Sahu et al. [90]. This could be attributed to the skeletal muscle protein and lipid breakdown in adipose tissue [91]. Hence, the coadministration of propolis (Gent+Pr), honey (Gent+H), and especially their combination (Gent+Pr+H) prevents markedly the final body weight loss. Both the control, propolis, and honey groups showed similar weights. The body weight change was accompanied by the alteration of the liver and kidney weights. The data (Table 6) showed that the daily administration of propolis or honey to normal rats does not affect the organs' relative weight. The data illustrates that the intraperitoneal injections of gentamicin (120 mg/kg BW/day, i.p.) for 10 consecutive days lead to a significant increase of organs' relative weight in comparison with the normal group, and this goes in hand with the findings of Laaroussi et al. [92]. The relative organ weight increase might be related to the inflammatory and oxidative stress effects of gentamicin overuse. GENT-rats simultaneously cotreated with honey alone, propolis alone, or both showed a significant decrease in the liver and kidney relative weights, with the best data displayed by the combined coadministration (Gent+Pr+H). This is consistent with the result of Nassar and coworkers, in which propolis, honey, and their mixture prevent the liver, kidney, heart, and lung relative weight decrease [93].

## 5. Conclusion

Phytochemical analysis of bee honey and propolis extracts showed the presence of several natural antioxidants belonging to different chemical groups: flavonoids, phenolic acids, flavonols, and stilbenes. These may be responsible for the documented efficacy of bee honey and propolis extracts in protecting biochemical characteristics and enzymatic activities of kidneys and liver tissues from alterations induced by gentamicin. Overall, daily intake of propolis and/or honey could offer promising protective effects on hepatic and renal functions, as well as maintaining the redox homeostasis. Further investigations would be needed to evaluate and understand the exact mechanism by which these extracts, possibly phenolic compounds, improve gentamicin-cause oxidative stress and hepatorenal injuries.

## Figures and Tables

**Table 1:** Phytochemical constituents and antioxidant activities of honey and propolis extracts.

Samples	Polyphenols (mg GAE/g)	Flavonoids (mg QE/g)	DPPH IC_50_ (mg/ml)	ABTS IC_50_ (mg/ml)	FRAP (mmol Fe^2+^/g)
Honey	2.08 ± 0.04	0.74 ± 0.01	9.78 ± 0.95	4.91 ± 0.12	0.025 ± 0.00
Propolis	76.00 ± 2.15	42.22 ± 0.96	0.15 ± 0.01	0.08 ± 0.02	2.15 ± 0.04
Honey+propolis	73.98 ± 4.27	44.01 ± 0.13	0.13 ± 0.02	0.41 ± 0.05	2.27 ± 0.09

DPPH: 2,2-diphenyl-1-picryl-hydrazyl-hydrate; ABTS: 2,2′-azino-bis(3-ethylbenzothiazoline-6-sulfonic acid) diammonium salt; FRAP: ferric reducing antioxidant power.

**Table 2:** Phenolic compound identification and quantification of honey and propolis extracts.

Compounds	Propolis	Honey
Vanillic acid	8.61 ± 0.30	2.90 ± 0.01
o-Coumaric acid	11.44 ± 4.63	n.d.
Ferulic acid	18.84 ± 0.21	8.35 ± 0.01
Ellagic acid	28.55 ± 1.99	5.09 ± 0.02
Naringin	35.78 ± 4.10	n.d.
Hesperidin	417.18 ± 50.0	n.d.
Apigenin	38.39 ± 2.60	n.d.
Cinnamic acid	n.d.	4.25 ± 0.01
Resveratrol	116.89 ± 12.7	n.d.
Rosmarinic acid	470.35 ± 52.00	n.d.
Rutin	12.40 ± 0.42	n.d.
Chlorogenic acid	16.11 ± 0.12	7.06 ± 0.11
Quercetin	12.02 ± 0.13	n.d.
Kaempferol	21.90 ± 1.60	n.d.
Gallic acid	n.d.	30.06 ± 0.23
Total	1208.5	57.7

Values of phenolic compounds are expressed as concentration (mg/l) mean ± SD of 3 experiments. n.d.: not detected.

**Table 3:** Effect of propolis, honey, or their combination on gentamicin-induced changes in kidney antioxidant enzymes, proteins, and MDA concentrations.

Experimental groups	Variables in the kidney				
	CAT (*μ*mol H_2_O_2_/min/mg prt)	GSH (*μ*g/mg prt)	GPx (nmol GSH/min/mg prt)	Proteins (mg/g tissue)	MDA (nmol/g tissue)
DW	12.08 ± 0.31	95.03 ± 5.32	15.72 ± 0.28	4.16 ± 0.07	62.46 ± 3.24
Pr	12.19 ± 0.23	96.76 ± 4.84	15.12 ± 0.37	4.18 ± 0.19	62.86 ± 8.65
H	12.11 ± 0.41	97.89 ± 5.11	15.17 ± 0.31	4.82 ± 0.13	59.97 ± 6.37
Gent	7.21 ± 0.41^a^^∗∗∗^	40.10 ± 2.55^a^^∗∗∗^	9.67 ± 0.22^a^^∗∗∗^	2.96 ± 0.11^a^^∗∗∗^	112.54 ± 9.10^a^^∗∗∗^
Gent+Pr	10.96 ± 0.84^b^^∗∗∗^	84.69 ± 4.09^a^^∗^^b^^∗∗∗^	14.17 ± 0.18^a^^∗^^b^^∗∗∗^	4.09 ± 0.09^b^^∗∗∗^	82.59 ± 11.02^b^^∗^
Gent+H	9.54 ± 0.69^a^^∗∗∗^^b^^∗∗^	79.80 ± 4.97^a^^∗^^b^^∗∗^	13.02 ± 0.19^a^^∗∗^^b^^∗∗∗^	5.12 ± 0.018^a^^∗∗∗^^b^^∗∗∗^	85.77 ± 10.96^b^^∗^
Gent+Pr+H	11.41 ± 0.39^b^^∗∗∗^^d^^∗∗^	89.24 ± 7.45^b^^∗∗∗^^d^^∗^	13.14 ± 0.30^a^^∗^^b^^∗∗∗^^c^^∗^	4.22 ± 0.24^b^^∗∗∗^^d^^∗∗∗^	79.71 ± 8.15^b^^∗∗^

CAT: catalase; GSH: glutathione; GPx: glutathione peroxidase; MDA: malondialdehyde. The results were presented as mean ± SD of 6 rats; DW: distilled water; Pr: propolis; H: honey; Gent: gentamicin. ^a^Comparison between the DW group and all groups; ^b^comparison between the Gent group and gentamicin cotreated groups; ^c^comparison between the Gent+Pr group and the Gent+Pr+H group; ^d^comparison between the Gent+H group and the Gent+Pr+H group. ^∗^*p* < 0.05, ^∗∗^*p* < 0.01, and ^∗∗∗^*p* < 0.001.

**Table 4:** Effect of propolis, honey, or their combination on gentamicin-induced changes in enzymatic antioxidants hepatic levels, proteins, and MDA levels.

Experimental groups	Variables in the liver				
	CAT (*μ*mol H_2_O_2_/min/mg prt)	GSH (*μ*g/mg prt)	GPx (nmol GSH/min/mg prt)	Proteins (mg/g tissue)	MDA (nmol/g tissue)
DW	15.74 ± 1.02	98.32 ± 3.42	8.09 ± 0.83	6.63 ± 0.19	45.20 ± 2.12
Pr	16.00 ± 1.30	99.91 ± 4.07	8.90 ± 0.58	6.67 ± 0.75	44.12 ± 0.85
H	15.42 ± 1.02	96.30 ± 2.64	9.12 ± 0.47	6.52 ± 0.37	45.81 ± 1.26
Gent	11.85 ± 1.08^a^^∗∗^	44.71 ± 2.91^a^^∗∗^	5.40 ± 0.89^a^^∗∗∗^	4.01 ± 0.58^a^^∗^	62.42 ± 1.34^a^^∗∗∗^
Gent+Pr	15.07 ± 0.87^b^^∗^	80.83 ± 1.19^b^^∗∗∗^	7.86 ± 0.81^a^^∗∗^^b^^∗^	6.15 ± 0.72^b^^∗^	50.64 ± 1.48^b^^∗∗^
Gent+H	14.65 ± 0.92^b^^∗^	83.75 ± 2.78^b^^∗∗∗^	7.11 ± 0.88^a^^∗^	6.03 ± 0.73^b^^∗^	51.23 ± 0.98^b^^∗∗^
Gent+Pr+H	15.33 ± 0.72^b^^∗∗∗^	88.40 ± 1.39^b^^∗∗∗^^d^^∗^	8.11 ± 0.55^b^^∗^	6.31 ± 0.79^b^^∗^	48.12 ± 1.08^b^^∗∗∗^^c^^∗^

CAT: catalase; GSH: glutathione; GPx: glutathione peroxidase; MDA: malondialdehyde. The results were presented as mean ± SD of 6 rats; DW: distilled water; Pr: propolis; H: honey; Gent: gentamicin. ^a^Comparison between the DW group and all groups; ^b^comparison between the Gent group and gentamicin cotreated groups; ^c^comparison between the Gent+Pr group and the Gent+Pr+H group; ^d^comparison between the Gent+H group and the Gent+Pr+H group. ^∗^*p* < 0.05, ^∗∗^*p* < 0.01, and ^∗∗∗^*p* < 0.001.

**Table 5:** Comparison between kidney and liver oxidative/antioxidant status.

Parameter	Gent					Gent+PR				
	Kidney		Liver			Kidney		Liver		
	Effect	%A	Effect	%A	*p*	Effect	%B	Effect	%B	*p*
CAT	Decrease	40.31	Decrease	24.67^a^	0.001	Increase	52.10	Increase	27.15^b^	0.001
GSH	Decrease	57.80	Decrease	54.52^a^	0.005	Increase	111.87	Increase	80.78^b^	0.001
GPx	Decrease	38.43	Decrease	40.56^a^	0.006	Increase	46.46	Increase	36.18^b^	0.002
Proteins	Decrease	28.58	Decrease	39.44^a^	0.002	Increase	37.53	Increase	53.27^b^	0.001
MDA	Increase	80.27	Increase	38.27^a^	0.001	Decrease	26.61	Decrease	18.87^b^	0.009
Parameter	Gent+H					Gent+PR+H				
	Kidney		Liver			Kidney		Liver		
	Effect	%C	Effect	%C	*p*	Effect	%D	Effect	%D	*p*
CAT	Increase	32.36	Increase	23.58^c^	0.001	Increase	58.29	Increase	29.32^d^	0.001
GSH	Increase	99.00	Increase	87.31^c^	0.003	Increase	122.54	Increase	97.80^d^	0.001
GPx	Increase	34.65	Increase	31.56^c^	0.002	Increase	35.79	Increase	50.18^d^	0.001
Proteins	Increase	72.08	Increase	50.29^c^	0.001	Increase	41.92	Increase	57.09^d^	0.001
MDA	Decrease	23.82	Decrease	18.00	0.031	Decrease	29.21	Decrease	24.36	0.171

%A: percentage of changes from the control induced by GENT administration; %B: percentage of changes from the GENT caused by propolis administration: %C: percentage of changes from the GENT caused by honey administration; %D: percentage of changes from the GENT caused by propolis+honey administration. ^a^*p* < 0.05: statistically significant as compared to the effect GENT on kidney oxidative parameters; ^b^*p* < 0.05: statistically significant as compared to the effect GENT+PR on kidney oxidative parameters; ^c^*p* < 0.05: statistically significant as compared to the effect GENT+H on kidney oxidative parameters; ^d^*p* < 0.05: statistically significant as compared to the effect GENT+PR+H on kidney oxidative parameters.

**Table 6:** The effect of propolis, honey, or their combination on the gentamicin-induced body and organ weight changes.

Variables	Experimental groups						
	DW	Pr	H	Gent	Gent+Pr	Gent+H	Gent+Pr+H
Initial BW (g)	161.81 ± 5.8	159.45 ± 4.15	164.26 ± 6.01	164.12 ± 4.91	160.16 ± 5.21	158.93 ± 4.81	163.76 ± 6.45
Final BW (g)	201.76 ± 3.23	195.88 ± 4.62	223.98 ± 4.36	189.99 ± 4.32	191.27 ± 3.18	193.99 ± 3.48	200.82 ± 3.95
BW gain (g)	39.95 ± 0.51	36.43 ± 1.32	59.72 ± 5.14^a^^∗∗∗^	25.87 ± 2.15^a^^∗∗∗^	31.11 ± 1.87^a^^∗^^b^^∗^	35.06 ± 2.56^b^^∗∗∗^	37.06 ± 2.25^b^^∗∗∗^^c^^∗^
Liver weight (g)	7.11 ± 0.06	6.88 ± 0.08	7.95 ± 0.04	5.34 ± 0.01^a^^∗^	6.20 ± 0.06^b^^∗^	6.19 ± 0.08^b^^∗^	6.92 ± 0.04^b^^∗^
Kidney weight (g)	1.89 ± 0.05	1.86 ± 0.02	2.06 ± 0.05^a^^∗^	1.58 ± 0.02^a^^∗∗∗^	1.67 ± 0.03^a^^∗^^b^^∗∗^	1.71 ± 0.02^a^^∗^^b^^∗∗^	1.85 ± 0.03^b^^∗∗∗^^c^^∗∗^^d^^∗∗^
LRW (g/100 g BW)	3.52 ± 0.09	3.51 ± 0.03	3.55 ± 0.01	2.81 ± 0.03^a^^∗∗∗^	3.35 ± 0.01^a^^∗∗^^b^^∗∗∗^	3.19 ± 0.04^a^^∗∗∗^^b^^∗∗^	3.45 ± 0.07^b^^∗∗∗^^d^^∗∗^
KRW (g/100 g BW)	0.94 ± 0.04	0.95 ± 0.01	0.92 ± 0.02	0.83 ± 0.01^a^^∗∗^	0.90 ± 0.03^b^^∗∗^	0.88 ± 0.02^a^^∗^^b^^∗^	0.92 ± 0.01^b^^∗∗^^d^^∗^

BW: body weight; LRW: liver relative weight; KRW: kidney relative weight. ^a^Comparison between the DW group and all groups; ^b^comparison between the Gent group and gentamicin cotreated groups; ^c^comparison between the Gent+Pr group and the Gent+Pr+H group; ^d^comparison between the Gent+H group and the Gent+Pr+H group. ^∗^*p* < 0.05, ^∗∗^*p* < 0.01, and ^∗∗∗^*p* < 0.001.

## Data Availability

The data used to support the findings of this study are included within the article.

## References

[1] Mohamadi Yarijani Z., Najafi H., Shackebaei D., Madani S. H., Modarresi M., Jassemi S. V. (2019). Amelioration of renal and hepatic function, oxidative stress, inflammation and histopathologic damages by *Malva sylvestris* extract in gentamicin induced renal toxicity. *Biomedicine & Pharmacotherapy*.

[2] Rodriguez-Barbero A., López-Novoa J. M., Arévalo M. (1997). Involvement of platelet-activating factor in gentamicin nephrotoxicity in rats. *Experimental Nephrology*.

[3] Galaly S. R., Ahmed O. M., Mahmoud A. M. (2014). Thymoquinone and curcumin prevent gentamicin-induced liver injury by attenuating oxidative stress, inflammation and apoptosis. *Journal of Physiology and Pharmacology: An Official Journal of the Polish Physiological Society*.

[4] He L., Peng X., Zhu J. (2015). Protective effects of curcumin on acute gentamicin-induced nephrotoxicity in rats. *Canadian Journal of Physiology and Pharmacology*.

[5] Banday A. A., Farooq N., Priyamvada S., Yusufi A. N. K., Khan F. (2008). Time dependent effects of gentamicin on the enzymes of carbohydrate metabolism, brush border membrane and oxidative stress in rat kidney tissues. *Life Sciences*.

[6] Sanchez-Gonzalez P. D., Lopez-Hernandez F. J., Perez-Barriocanal F., Morales A. I., Lopez-Novoa J. M. (2011). Quercetin reduces cisplatin nephrotoxicity in rats without compromising its anti-tumour activity. *Nephrology Dialysis Transplantation*.

[7] Tugcu V., Ozbek E., Tasci A. I. (2006). Selective nuclear factor kappa-B inhibitors, pyrolidium dithiocarbamate and sulfasalazine, prevent the nephrotoxicity induced by gentamicin. *BJU International*.

[8] Volpini R. A., Balbi A. P. C., Costa R. S., Coimbra T. M. (2006). Increased expression of p38 mitogen-activated protein kinase is related to the acute renal lesions induced by gentamicin. *Brazilian Journal of Medical and Biological Research*.

[9] Bankova V., Popova M., Trusheva B. (2016). New emerging fields of application of propolis. *Macedonian Journal of Chemistry and Chemical Engineering*.

[10] Viel C., Doré J. C. (2003). Histoire et emplois du miel, de l’hydromel et des produits de la ruche. *Revue d'Histoire de la Pharmacie*.

[11] Laaroussi H., Bouddine T., Bakour M., Ousaaid D., Lyoussi B. (2020). Physicochemical properties, mineral content, antioxidant activities, and microbiological quality of Bupleurum spinosum Gouan honey from the middle atlas in Morocco. *Journal of Food Quality*.

[12] Saxena S., Gautam S., Sharma A. (2010). Physical, biochemical and antioxidant properties of some Indian honeys. *Food Chemistry*.

[13] Pasupuleti V. R., Sammugam L., Ramesh N., Gan S. H. (2017). Honey, Propolis, and Royal Jelly: A Comprehensive Review of Their Biological Actions and Health Benefits. *Oxidative Medicine and Cellular Longevity*.

[14] Thawley A. (1969). The components of honey and their effects on its properties: a review. *Bee World*.

[15] Sforcin J. M., Bankova V. (2011). Propolis: is there a potential for the development of new drugs?. *Journal of Ethnopharmacology*.

[16] Wagh V. D. (2013). Propolis: a wonder bees product and its pharmacological potentials. *Advances in Pharmacological Sciences*.

[17] Okail H. A., Ibrahim A. S., Badr A. H. (2020). The protective effect of propolis against aluminum chloride-induced hepatorenal toxicity in albino rats. *The Journal of Basic and Applied Zoology*.

[18] Laaroussi H., Ferreira-Santos P., Genisheva Z. (2021). Unraveling the chemical composition, antioxidant, *α*-amylase and *α*-glucosidase inhibition of Moroccan propolis. *Food Bioscience*.

[19] Martinello M., Mutinelli F. (2021). Antioxidant activity in bee products: a review. *Antioxidants*.

[20] al-Hatamleh M. A. I., Boer J. C., Wilson K. L., Plebanski M., Mohamud R., Mustafa M. Z. (2020). Antioxidant-based medicinal properties of stingless bee products: recent progress and future directions. *Biomolecules*.

[21] Phan M. A. T., Paterson J., Bucknall M., Arcot J. (2018). Interactions between phytochemicals from fruits and vegetables: effects on bioactivities and bioavailability. *Critical Reviews in Food Science and Nutrition*.

[22] Bakour M., Laaroussi H., el menyiy N., Elaraj T., el ghouizi A., Lyoussi B. (2021). The Beekeeping State and Inventory of Mellifero-Medicinal Plants in the North- Central of Morocco. *The Scientific World Journal*.

[23] Ferreira-Santos P., Genisheva Z., Pereira R. N., Teixeira J. A., Rocha C. M. R. (2019). Moderate electric fields as a potential tool for sustainable recovery of phenolic compounds fromPinus pinasterBark. *ACS Sustainable Chemistry & Engineering*.

[24] Kong K. W., Mat-Junit S., Aminudin N., Ismail A., Abdul-Aziz A. (2012). Antioxidant activities and polyphenolics from the shoots of *Barringtonia racemosa* (L.) Spreng in a polar to apolar medium system. *Food Chemistry*.

[25] Ozbek E., Cekmen M., Ilbey Y. O., Simsek A., Polat E. C., Somay A. (2009). Atorvastatin prevents gentamicin-induced renal damage in rats through the inhibition of p38-MAPK and NF-kB pathways. *Renal Failure*.

[26] el-haskoury R., al-Waili N., Kamoun Z., Makni M., al-Waili H., Lyoussi B. (2018). Antioxidant activity and protective effect of carob honey in CCl_4_-induced kidney and liver injury. *Archives of Medical Research*.

[27] El Menyiy N., Al Waili N., Bakour M., Al-Waili H., Lyoussi B. (2016). Protective effect of propolis in proteinuria, crystaluria, nnephrotoxicity and hepatotoxicity induced by ethylene glycol ingestion. *Archives of Medical Research*.

[28] Bakour M., Hammas N., Laaroussi H. (2021). Moroccan bee bread improves biochemical and histological changes of the brain, liver, and kidneys induced by titanium dioxide nanoparticles. *BioMed Research International*.

[29] Aebi H. (1984). [13] Catalase in vitro. *Methods in Enzymology*.

[30] Flohé L., Günzler W. A. (1984). Assays of glutathione peroxidase. *Methods in Enzymology*.

[31] Ellman G. L. (1959). Tissue sulfhydryl groups. *Archives of Biochemistry and Biophysics*.

[32] Kassan M., Montero M. J., Sevilla M. A. (2009). Chronic treatment with pravastatin prevents early cardiovascular changes in spontaneously hypertensive rats. *British Journal of Pharmacology*.

[33] Zhang H., Tsao R. (2016). Dietary polyphenols, oxidative stress and antioxidant and anti-inflammatory effects. *Current Opinion in Food Science*.

[34] Touzani S., al-Waili N., el Menyiy N. (2018). Chemical analysis and antioxidant content of various propolis samples collected from different regions and their impact on antimicrobial activities. *Asian Pacific Journal of Tropical Medicine*.

[35] da Graça Miguel M., Doughmi O., Aazza S., Antunes D., Lyoussi B. (2014). Antioxidant, anti-inflammatory and acetylcholinesterase inhibitory activities of propolis from different regions of Morocco. *Food Science and Biotechnology*.

[36] do Nascimento T. G., dos Santos Arruda R. E., da Cruz Almeida E. T. (2019). Comprehensive multivariate correlations between climatic effect, metabolite- profile, antioxidant capacity and antibacterial activity of Brazilian red propolis metabolites during seasonal study. *Sci. Rep.*.

[37] Svečnjak L., Marijanović Z., Okińczyc P., Marek Kuś P., Jerković I. (2020). Mediterranean propolis from the Adriatic Sea islands as a source of natural antioxidants: comprehensive chemical biodiversity determined by GC-MS, FTIR-ATR, UHPLC-DAD-QqTOF-MS, DPPH and FRAP assay. *Antioxidants*.

[38] Petretto G. L., Tuberoso C. I. G., Fenu M. A., Rourke J. P., Belhaj O., Pintore G. (2017). Antioxidant activity, color chromaticity coordinates, and chemical characterization of monofloral honeys from Morocco. *International Journal of Food Properties*.

[39] Maringgal B., Hashim N., Tawakkal I., Mohamed M., Shukor N. I. A. (2019). Phytochemical compositions and antioxidant activities of malaysian stingless bee honey. *Pertanika Journal of Science and Technology*.

[40] Lazarević K. B., Andrić F., Trifković J., Tešić Ž., Milojković-Opsenica D. (2012). Characterisation of Serbian unifloral honeys according to their physicochemical parameters. *Food Chemistry*.

[41] Kıvrak Ş., Kıvrak İ. (2017). Assessment of phenolic profile of Turkish honeys. *International Journal of Food Properties*.

[42] Mahmoodi-Khaledi E., Lozano-Sánchez J., Bakhouche A., Habibi-Rezaei M., Sadeghian I., Segura-Carretero A. (2017). Physicochemical properties and biological activities of honeys from different geographical and botanical origins in Iran. *European Food Research and Technology*.

[43] Iacopini P., Baldi M., Storchi P., Sebastiani L. (2008). Catechin, epicatechin, quercetin, rutin and resveratrol in red grape: Content, *in vitro* antioxidant activity and interactions. *Journal of Food Composition and Analysis*.

[44] Skroza D., Generalić Mekinić I., Svilović S., Šimat V., Katalinić V. (2015). Investigation of the potential synergistic effect of resveratrol with other phenolic compounds: a case of binary phenolic mixtures. *Journal of Food Composition and Analysis*.

[45] Jose S. P., Asha S., Krishnakumar I. M. (2017). Nephro-protective effect of a novel formulation of unopened coconut inflorescence sap powder on gentamicin induced renal damage by modulating oxidative stress and inflammatory markers. *Biomedicine & Pharmacotherapy*.

[46] Shahani S., Behzadfar F., Jahani D., Ghasemi M., Shaki F. (2017). Antioxidant and anti-inflammatory effects of Nasturtium officinale involved in attenuation of gentamicin-induced nephrotoxicity. *Toxicology Mechanisms and Methods*.

[47] Marinho A. D., Silveira J. A. M., Chaves-Filho A. J. M. (2020). Protective effects of a lipid transfer protein isolated from Morinda citrifolia seeds in gentamicin-induced nephrotoxicity in rats. *Revista Brasileira de Farmacognosia*.

[48] Ali B. H., al Za’abi M., Blunden G., Nemmar A. (2011). Experimental gentamicin nephrotoxicity and agents that modify it: a mini-review of recent research. *Basic & Clinical Pharmacology & Toxicology*.

[49] Lakhera A., Ganeshpurkar A., Bansal D., Dubey N. (2015). Chemopreventive role of Coriandrum sativum against gentamicin-induced renal histopathological damage in rats. *Interdisciplinary Toxicology*.

[50] Aldahmash B. A., el-Nagar D. M., Ibrahim K. E. (2016). Efectos renoprotectores del propoleo sobre la toxicidad renal aguda inducida por gentamicina en ratones albinos suizos. *Nefrología*.

[51] Ferreira-Santos P., Zanuso E., Genisheva Z., Rocha C. M. R., Teixeira J. A. (2020). Green and sustainable valorization of bioactive phenolic compounds from pinus by-products. *Molecules*.

[52] Tavafi M., Ahmadvand H., Tamjidipour A., Hasanvand A. (2019). Rosmarinic acid ameliorates renal ischemia reperfusion damage in rats. *Journal of Nephropharmacology*.

[53] Domitrović R., Potočnjak I., Crnčević-Orlić Ž., Škoda M. (2014). Nephroprotective activities of rosmarinic acid against cisplatin-induced kidney injury in mice. *Food and Chemical Toxicology*.

[54] Mohammadi S., Karimi J., Tavilani H., Khodadadi I., Mohseni R., Hashemnia M. (2020). Resveratrol downregulates TGF-*β*1 and Smad3 expression and attenuates oxidative stress in CCl4-induced kidney damage in rats. *Asian Pacific Journal of Tropical Biomedicine*.

[55] Edeogu C., Kalu M. E., Famurewa A. C., Asogwa N. T., Onyeji G. N., Ikpemo K. O. (2020). Nephroprotective effect ofMoringa OleiferaSeed oil on gentamicin-induced nephrotoxicity in rats: biochemical evaluation of antioxidant, anti-inflammatory, and antiapoptotic pathways. *Journal of the American College of Nutrition*.

[56] Kovács E., Savopol T., Iordache M. M. (2012). Interaction of gentamicin polycation with model and cell membranes. *Bioelectrochemistry*.

[57] Walker P. D., Shah S. V. (1988). Evidence suggesting a role for hydroxyl radical in gentamicin-induced acute renal failure in rats. *The Journal of Clinical Investigation*.

[58] Collin F. (2019). Chemical basis of reactive oxygen species reactivity and involvement in neurodegenerative diseases. *International Journal of Molecular Sciences*.

[59] Ansari M. A., Raish M., Ahmad A. (2016). Sinapic acid mitigates gentamicin-induced nephrotoxicity and associated oxidative/nitrosative stress, apoptosis, and inflammation in rats. *Life Sciences*.

[60] Mahmoud A. M., Abd el-Ghafar O. A. M., Alzoghaibi M. A., Hassanein E. H. M. (2021). Agomelatine prevents gentamicin nephrotoxicity by attenuating oxidative stress and TLR-4 signaling, and upregulating PPAR*γ* and SIRT1. *Life Sciences*.

[61] Gaetke L. M., Chow-Johnson H. S., Chow C. K. (2014). Copper: toxicological relevance and mechanisms. *Archives of Toxicology*.

[62] Weyemi U., Lagente-Chevallier O., Boufraqech M. (2012). ROS-generating NADPH oxidase NOX4 is a critical mediator in oncogenic H-Ras- induced DNA damage and subsequent senescence. *Oncogene*.

[63] Weyemi U., Dupuy C. (2012). The emerging role of ROS-generating NADPH oxidase NOX4 in DNA-damage responses. *Mutation Research*.

[64] Sedelnikova O. A., Redon C. E., Dickey J. S., Nakamura A. J., Georgakilas A. G., Bonner W. M. (2010). Role of oxidatively induced DNA lesions in human pathogenesis. *Mutation Research*.

[65] Shi Y., Liu Y. C., Zheng Y. F. (2019). Ethanol extract of Chinese propolis attenuates early diabetic retinopathy by protecting the blood–retinal barrier in streptozotocin-induced diabetic rats. *Journal of Food Science*.

[66] Biluca F. C., da Silva B., Caon T. (2020). Investigation of phenolic compounds, antioxidant and anti-inflammatory activities in stingless bee honey (*Meliponinae*). *Food Research International*.

[67] Nouri A., Heibati F., Heidarian E. (2021). Gallic acid exerts anti-inflammatory, anti-oxidative stress, and nephroprotective effects against paraquat-induced renal injury in male rats. *Naunyn-Schmiedeberg's Archives of Pharmacology*.

[68] Siddiqi A., Saidullah B., Sultana S. (2018). Anti-carcinogenic effect of hesperidin against renal cell carcinoma by targeting COX-2/PGE2 pathway in Wistar rats. *Environmental Toxicology*.

[69] Hegazy A. M., Abdel-Azeem A. S., Zeidan H. M., Ibrahim K. S., Sayed E. M. E. (2018). Hypolipidemic and hepatoprotective activities of rosemary and thyme in gentamicin-treated rats. *Human & Experimental Toxicology*.

[70] Bakour M., Soulo N., Hammas N. (2018). The antioxidant content and protective effect of argan oil and Syzygium aromaticum essential oil in hydrogen peroxide-induced biochemical and histological changes. *International Journal of Molecular Sciences*.

[71] Peter B., Wartena M., Kampinga H. H., Konings A. W. T. (1992). Role of lipid peroxidation and DNA damage in paraquat toxicity and the interaction of paraquat with ionizing radiation. *Biochemical Pharmacology*.

[72] Korashy H. M., Attafi I. M., Famulski K. S. (2017). Gene expression profiling to identify the toxicities and potentially relevant human disease outcomes associated with environmental heavy metal exposure. *Environmental Pollution*.

[73] Manubolu M., Goodla L., Ravilla S. (2014). Protective effect of Actiniopteris radiata (Sw.) Link. against CCl4 induced oxidative stress in albino rats. *Journal of Ethnopharmacology*.

[74] Abd-Elhakim Y. M., Moselhy A. A. A., Aldhahrani A. (2021). Protective effect of curcumin against sodium salicylate-induced oxidative kidney damage, nuclear factor-kappa dysregulation, and apoptotic consequences in rats. *Antioxidants.*.

[75] Galal R. M., Zaki H. F., Seif el-Nasr M. M., Agha A. M. (2012). Potential protective effect of honey against paracetamol-induced hepatotoxicity. *Archives of Iranian Medicine*.

[76] Kaya E., Yılmaz S., Ceribasi S. (2019). Protective role of propolis on low and high dose furan-induced hepatotoxicity and oxidative stress in rats. *Journal of Veterinary Research*.

[77] Diniz D. P., Lorencini D. A., Berretta A. A. (2020). Antioxidant Effect of Standardized Extract of Propolis (EPP-AF®) in Healthy Volunteers: A “Before and After” Clinical Study. *Evidence-based Complementary and Alternative Medicine*.

[78] Ebeid S. A., Abd el Moneim N. A., el-Benhawy S. A., Hussain N. G., Hussain M. I. (2016). Assessment of the radioprotective effect of propolis in breast cancer patients undergoing radiotherapy. New perspective for an old honey bee product. *Journal of Radiation Research and Applied Science*.

[79] Gamulin M., Garaj-Vrhovac V., Kopjar N. (2007). Evaluation of DNA damage in radiotherapy-treated cancer patients using the alkaline comet assay. *Collegium Antropologicum*.

[80] Haza A. I., Morales P. (2013). Spanish honeys protect against food mutagen-induced DNA damage. *Journal of the Science of Food and Agriculture*.

[81] Robards K., Prenzler P. D., Tucker G., Swatsitang P., Glover W. (1999). Phenolic compounds and their role in oxidative processes in fruits. *Food Chemistry*.

[82] Mahmoud A. M., Hussein O. E., Hozayen W. G., Bin-Jumah M., Abd el-Twab S. M. (2020). Ferulic acid prevents oxidative stress, inflammation, and liver injury via upregulation of Nrf2/HO-1 signaling in methotrexate-induced rats. *Environmental Science and Pollution Research*.

[83] Reyes-Esparza1 J., Escutia-Gutiérrez R., Garcia-Vázquez F. (2019). Gallic acid produces hepatoprotection by modulating EGFR expression and phosphorylation in induced preneoplastic liver foci in rats. *Open Journal of Gastroenterology and Hepatology*.

[84] Ren J., Lu Y., Qian Y., Chen B., Wu T., Ji G. (2019). Recent progress regarding kaempferol for the treatment of various diseases (Review). *Experimental and Therapeutic Medicine*.

[85] Dkhil M. A., Abdel Moneim A. E., Bauomy A. A., Khalil M., al-Shaebi E. M., al-Quraishy S. (2020). Chlorogenic acid prevents hepatotoxicity in arsenic-treated mice: role of oxidative stress and apoptosis. *Molecular Biology Reports*.

[86] Hidalgo M., Sánchez-Moreno C., de Pascual-Teresa S. (2010). Flavonoid-flavonoid interaction and its effect on their antioxidant activity. *Food Chemistry*.

[87] Zhou B., Miao Q., Yang L., Liu Z. L. (2005). Antioxidative effects of flavonols and their glycosides against the free-radical-induced peroxidation of linoleic acid in solution and in micelles. *Chemistry - A European Journal*.

[88] Arias N., Macarulla M. T., Aguirre L., Milton I., Portillo M. P. (2016). The combination of resveratrol and quercetin enhances the individual effects of these molecules on triacylglycerol metabolism in white adipose tissue. *European Journal of Nutrition*.

[89] Baranowska M., Koziara Z., Suliborska K. (2021). Interactions between polyphenolic antioxidants quercetin and naringenin dictate the distinctive redox-related chemical and biological behaviour of their mixtures. *Scientific Reports*.

[90] Sahu B. D., Kuncha M., Putcha U. K., Sistla R. (2013). Effect of metformin against cisplatin induced acute renal injury in rats: a biochemical and histoarchitectural evaluation. *Experimental and Toxicologic Pathology*.

[91] Whitehouse A. S., Smith H. J., Drake J. L., Tisdale M. J. (2001). Mechanism of attenuation of skeletal muscle protein catabolism in cancer cachexia by eicosapentaenoic acid. *Cancer Research*.

[92] Laaroussi H., Bakour M., Ousaaid D. (2020). Effect of antioxidant-rich propolis and bee pollen extracts against D-glucose induced Type 2 Diabetes in rats. *Food Research International*.

[93] Nassar A. M. K., Salim Y. M. M., Eid K. S. A. (2020). Ameliorative effects of honey, propolis, pollen, and royal jelly mixture against chronic toxicity of sumithion insecticide in white Albino rats. *Molecules*.

